# Evolution of winter molting strategies in European and North American migratory passerines

**DOI:** 10.1002/ece3.8047

**Published:** 2021-09-01

**Authors:** Claudie Pageau, Jared Sonnleitner, Christopher M. Tonra, Mateen Shaikh, Matthew W. Reudink

**Affiliations:** ^1^ Department of Biological Sciences Thompson Rivers University Kamloops BC Canada; ^2^ School of Environment and Natural Resources The Ohio State University Columbus Ohio USA; ^3^ Department of Mathematics & Statistics Thompson Rivers University Kamloops BC Canada

**Keywords:** molt, Nearctic, Passeriformes, phylogenetic analysis, Western Palearctic

## Abstract

Molt is critical for birds as it replaces damaged feathers and worn plumage, enhancing flight performance, thermoregulation, and communication. In passerines, molt generally occurs on the breeding grounds during the postbreeding period once a year. However, some species of migrant passerines that breed in the Nearctic and Western Palearctic regions have evolved different molting strategies that involve molting on the overwintering grounds. Some species forego molt on the breeding grounds and instead complete their prebasic molt on the overwintering grounds. Other species molt some or all feathers a second time (prealternate molt) during the overwintering period. Using phylogenetic analyses, we explored the potential drivers of the evolution of winter molts in Nearctic and Western Palearctic breeding passerines. Our results indicate an association between longer photoperiods and the presence of prebasic and prealternate molts on the overwintering grounds for both Nearctic and Western Palearctic species. We also found a relationship between prealternate molt and generalist and water habitats for Western Palearctic species. Finally, the complete prealternate molt in Western Palearctic passerines was linked to longer days on the overwintering grounds and longer migration distance. Longer days may favor the evolution of winter prebasic molt by increasing the time window when birds can absorb essential nutrients for molt. Alternatively, for birds undertaking a prealternate molt at the end of the overwintering period, longer days may increase exposure to feather‐degrading ultra‐violet radiation, necessitating the replacement of feathers. Our study underlines the importance of the overwintering grounds in the critical process of molt for many passerines that breed in the Nearctic and Western Palearctic regions.

## Graphical abstract

Our study explored the potential drivers of the evolution of winter molts in Nearctic and Western Palearctic breeding passerines. Using phylogenic analysis, we tested whether photoperiod, migration distance, NDVI of the breeding and overwintering grounds, habitat, and diet were associated with species undergoing a winter prebasic molt or a prealternate molt. Our study suggests that the evolution of winter molt strategies in Nearctic and Western Palearctic migratory passerines was likely driven by multiple factors, but photoperiod, migration distance, and overwintering ground conditions are particularly important.

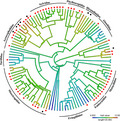

## 1 INTRODUCTION

Molt, the systematic replacement of old feathers by new ones, is critical for birds; fresh, high‐quality plumage enhances flight performance, thermoregulation, visual communication, and attractiveness (Gill, 2006). Hence, the timing, location, and number of molts are critical for birds to grow feathers of sufficient quality and maintain relatively fresh plumage throughout the annual cycle. Most migratory passerines (Order Passeriformes) that breed in the Nearctic and Western Palearctic regions undergo only one complete molt (*i.e*., all feathers are replaced) during the annual cycle (Humphrey & Parkes, 1959; Jenni & Winkler, 2020; Pyle, 1997). This molt generally occurs after breeding on the breeding grounds and is termed the prebasic molt for adult birds (Humphrey & Parkes, 1959). However, when and where the prebasic molt occurs can be quite variable. For example, some species or populations employ a strategy in which there is a temporal overlap between molt and migration (molt‐migration; Tonra & Reudink, 2018). This is especially common in western North America where many passerine species depart their arid breeding grounds to molt in the highly productive Mexican monsoon region prior to completing their southward migration (Pageau et al., 2020; Pyle et al., 2009; Rohwer et al., 2005). Other species migrate prior to molt and undergo the prebasic molt on the overwintering grounds (Barta et al., 2008; De la Hera et al., 2012; Kiat et al., 2019). Molt on the breeding grounds has been reported to be the ancestral state of the prebasic molt, and other strategies evolved later (Svensson & Hedenström, 1999), likely in response to environmental and life‐history trade‐offs (Pageau, Tonra, et al., 2020).

In addition to prebasic molt, some species undergo a second molt, termed prealternate and resulting in alternate plumage (Humphrey & Parkes, 1959), at some point during the annual cycle, usually in late winter/early spring prior to breeding, although prealternate molt‐migration has also been documented (e.g., Wright et al., 2018). Generally, this molt is partial (*i.e*., not all feathers are replaced) and often only involves body feathers (Jenni & Winkler, 2020). However, some migratory passerines undergo a complete prealternate molt, replacing all their feathers on the overwintering grounds prior to fall migration (e.g., *Dolichonyx oryzivorus* Renfrew et al., 2011, *Phylloscopus trochilus* Underhill et al., 1992). The presence of a complete prealternate molt during the annual cycle is rare among Nearctic migrant passerines, but more common among Western Palearctic passerines (Jenni & Winkler, 2020; Renfrew et al., 2011). Generally, species undergoing a complete prealternate molt in the Palearctic have an incomplete prebasic molt. Rarely will passerines, both in the Nearctic and in the Palearctic, have two complete molts during the annual cycle (Renfrew et al., 2011). This extensive variation in molt strategies among migratory passerines begs the question: Which life‐history characteristics and/or environmental factors have driven the evolution of these different molt strategies?

Pageau, Tonra, et al. (2020) studied the evolution of molt‐migration in North America using phylogenetic analysis to correct for the nonindependence among species. The results indicated the importance of the aridity of the breeding grounds and migration distance as potential drivers of the evolution of molt‐migration. Phylogenetic analyses are important for the study of evolutionary processes to correct for the nonindependence among species (Ives & Zhu, 2006), but they have not been used to look at the evolution of winter molt strategies in passerines. In a recent study of the Nearctic–Neotropical Family Parulidae, Terrill et al. (2020) concluded that structural needs driven by feather damage during the annual cycle drive the evolution of prealternate molts. However, the extent to which this is the case across the diverse molt strategies of passerines as a whole remains unknown.

Although phylogenetic analyses have not been conducted yet to study winter molts in all Passeriformes, potential drivers of the evolution of the prebasic molt on the overwintering grounds have been hypothesized (but see Svensson & Hedenström, 1999; Terrill et al., 2020 for studies on particular families). Barta et al. (2008) created models that linked winter molt in migratory birds with food seasonality; lack of resources at the end of the summer combined with an abundance of resources on the overwintering grounds during winter could have led to the evolution of winter molt (see also Remisiewicz et al., 2019 **)**. This would particularly be the case for Western Palearctic species migrating to sub‐Saharan overwintering grounds that are productive during the northern fall/beginning of winter because of the rainy season at these locations (Jenni & Winkler, 2020; Kiat et al., 2019). Molting on the overwintering grounds after migration would also occur most often in longer‐distance migrants; longer migration distance would impose a time constraint between breeding and migration and, thus, could favor molt somewhere other than breeding grounds (Kjellén, 1994; Leu & Thompson, 2002). De la Hera et al. (2012) recorded molt duration of 98 Nearctic passerines and found that migrant species molting on the overwintering grounds have a molt duration as long as resident species molting on breeding grounds. However, migrant species molting on the breeding grounds have a shorter molt duration. Previous studies have indicated that a longer molt duration results in feathers of higher quality (e.g., Dawson et al., 2000; Griggio et al., 2009; Serra, 2001); however, de la Hera et al. (2012) observed the opposite. Thus, it could be more advantageous for long‐distance migrants to molt on the overwintering grounds where there are fewer time constraints, which would allow them to grow high‐quality feathers.

The presence of a second molt during the annual cycle may be favored due to rapid degradation of the feathers from UV exposure (Bergman, 1982; Jenni & Winkler, 2020, see studies by Svensson & Hedenström, 1999; Jiguet et al., 2019). Thus, species living in open habitats would be more affected by UV degradation and more likely to undergo a prealternate molt (Guallar et al., 2021; Pyle, 1998; Pyle & Kayhart, 2010). This would particularly be the case for some Western Palearctic migrants who winter in more sun‐exposed environments such as savannahs (Jones, 1995; Rohwer et al., 2005). For Nearctic migrant passerines, Froehlich et al. (2005) observed a link between a plant‐based diet and the presence of a prealternate molt. Complete prealternate molt is more common in Western Palearctic migrant passerines than Nearctic migrant passerines; only *Dolichonyx oryzivorus* (Renfrew et al., 2011) undergoes a complete prealternate molt among Nearctic migrant passerines which results in two complete molts during the annual cycle. Rohwer et al. (2005) proposed that a complete prealternate molt is rare among Nearctic migrant passerines because most species winter in moister, denser, and heavily shaded habitats with softer foliage which would result in relatively less damage to feathers compared with open habitats.

Here, we explore potential drivers behind the evolution of winter molts, specifically the prebasic and prealternate winter molts in Nearctic and Western Palearctic migratory passerines. We tested whether migration distance, aridity of the breeding and overwintering grounds, average photoperiod (i.e., average day length over a certain period at a particular location), diet, and habitat of the overwintering grounds were associated with the presence of winter molts. Based on previous studies, we predicted that the prebasic molt on the overwintering grounds will be associated with longer‐distance migrants for both Nearctic and Western Palearctic (Kjellén, 1994; Leu & Thompson, 2002). We also predicted that productive overwintering grounds would be an additional driver of the winter prebasic molt for Western Palearctic species (Barta et al., 2008; Remisiewicz et al., 2019). For the prealternate molt, we predicted that overwintering in open habitats and locations where the average photoperiod is longer are important factors associated with a second molt for both Nearctic and Western Palearctic migrant passerines because these species would be more exposed to UV radiation resulting in feather degradation (Bergman, 1982; Jenni & Winkler, 2020; Jiguet et al., 2019; Pyle, 1998; Svensson & Hedenström, 1999; Terrill et al., 2020). We also predicted that herbivorous Nearctic species would be associated with the presence of a prealternate molt (Froehlich et al., 2005).

## 2 MATERIALS AND METHODS

### 2.1 Species selection

We collected data for all species of Nearctic and Western Palearctic migratory passerines according to Birds of North America (now The Birds of the World, 2020) and Handbook of Western Palearctic Birds (Shirihai & Svensson, 2018) lists. For the Nearctic species, we only kept species with a breeding distribution located in Canada or USA (excluding Hawaii) and overwintering grounds in the Americas. For the Western Palearctic, we kept species with a breeding distribution located in Europe, northern Africa, or western Asia (we used 61°46′35.2″ as an approximate eastern boundary and the Sahara Desert as southern boundary) and with overwintering grounds in Africa, Europe, or western Asia. We excluded species that were not considered full migrants by IUCN (2020) in addition to *Pinicola enucleator*, *Galerida cristata*, *Corvus corone,* and *Cinclus cinclus* as they were not considered full migrant by Handbook of the Birds of the World (now The Birds of the World, 2020). We deleted any species from our dataset that lacks important data for our analyses. Our final data consisted of 183 species (including 6 subspecies) of Nearctic migratory passerines and for 116 species (including 2 subspecies) of Western Palearctic migratory passerines.

### 2.2 Classification molt strategies

We followed Humphrey and Parkes (1959) system to classify two types of molt: prebasic molt, which results in the basic plumage and generally occurs after breeding, and prealternate molt, which results in the alternate plumage and generally occurs before spring migration. For the prebasic molt, we classified if the species molted on their overwintering grounds or not as a binary variable (overwintering grounds = 1, not = 0; Table 1). Species undergoing molt‐migration or conducting a suspended prebasic molt were not considered as molting on the overwintering grounds (classified as 0). For prealternate molt, we recorded whether the molt was complete, partial, or absent. Thus, we created two binary response variables for prealternate molt: (A) presence or absence of prealternate molt and (B) complete prealternate molt or absence of a complete prealternate molt (Table 1). To categorize these molting strategies, we extracted molt information from species description in The Birds of the World (2020) for Nearctic passerines and the Handbook of Western Palearctic Birds (Shirihai & Svensson, 2018) for Western Palearctic passerines. When the molting accounts were unclear, vague, or lacking, we searched other sources to find or verify the information and used the most recent information (The Identification Guide to North American Birds, Pyle, 1997; and peer‐reviewed journal articles; Voelker & Rohwer, 1998; Butler et al., 2002; Rohwer et al., 2005; Butler et al., 2006; Pyle et al., 2009; Jahn et al., 2013). When variation in molting strategy among individuals of the same species was encountered, we used the predominant strategy to classify the species. For the case of *Tyrannus vociferans* and *Delichon urbicum*, we classified the species as having a prebasic molt on the overwintering grounds even though the species have been reported to molt both on the breeding and overwintering grounds equally. We recorded the reference used for each species in the data file.

**TABLE 1:** Number of species for Nearctic (total of 183 species) and Western Palearctic (total of 116 species) passerines performing different molting strategies: winter prebasic, prealternate, and complete prealternate molt

	Nearctic	Palearctic
Winter prebasic	13/183 (7.1%)	9/116 (7.8%)
Prealternate	87/183 (47.5%)	56/116 (48.3%)
Complete prealternate	2/183 (1.1%)	11/116 (9.5%)

### 2.3 Data collection of predictor variables

To classify the amount of primary productivity on the breeding and overwintering grounds, we calculated the normalized difference vegetation index (NDVI) using distribution maps of the breeding and overwintering grounds from BirdLife International Data Zone after filling out a request (2018). NDVI is a measure of live green vegetation and was used to indicate the aridity of the breeding grounds during the postbreeding period (1 July to 31 August) and the overwintering grounds during the nonbreeding period (15 September to 15 April). Data were available from the Application for Extracting and Exploring Analysis Ready Samples (AppEEARS Team, 2019). We extracted the NDVI values from 2000 to 2019 using the “Area Sample” function, chose the product MOD13A3.006 (Didan, 2015), selected “Native projection,” and calculated the mean value for each species. One hundred ninety‐eight distribution maps were not available from BirdLife International (2018) or unusable in AppEEARS. Instead, we created distribution maps as polygons using template maps from IUCN (2020). Methods for NDVI data collection followed those employed by Pageau, Tonra, et al. (2020).

Migration distance was approximated as the distance (megameter; Mm) from the centroid of the breeding distribution to the centroid of the wintering distribution using the package *Geosphere* (Hijmans, 2019) in R (R Core Team, 2020). Centroid values were calculated in decimal degrees using polygon maps provided by BirdLife International (2018) in ArcMap. One hundred seventy maps were unavailable from BirdLife International, and the centroid values were calculated by creating polygons in Google Earth pro using template maps from IUCN (2020) and processing the polygons in Earth Point (2020).

Average photoperiods at the overwintering grounds were measured using each species centroid on the National Research Council of Canada (2020) sunset/sunrise calculator. Photoperiods on the overwintering grounds were retrieved from 15 September to 15 April 2018/2019 and averaged for each species.

We retrieved the list of preferred breeding and nonbreeding habitats for each species from BirdLife Data Zone online database (November 2018). We used data mining on the webpage http://datazone.birdlife.org/species/factsheet/common_name‐scientific_name/details for this task, and spaces were replaced by the character “‐” on common and scientific names. All entries were checked for nomenclature inconsistencies. For our analysis, we categorized and simplified the type of habitat for the overwintering grounds by prioritizing major habitat and then suitable. We also categorized the habitat into four major categories: dense (forest, shrubland), open (grassland, savanna, open woodland, rocky areas), water (wetland, marine), and generalist. We classified species as generalist when two or more major habitats were encountered. Habitat classification followed methods employed by Pageau, Vale, et al. (2020).

Diet data were retrieved from Willman et al. (2014), and we classified the species into three categories: herbivore, omnivore, and invertivore. We could not include diet in the winter prebasic models as every winter molt species except one had a diet that mostly consists of invertebrates.

### 2.4 Phylogeny

**FIGURE 1:**
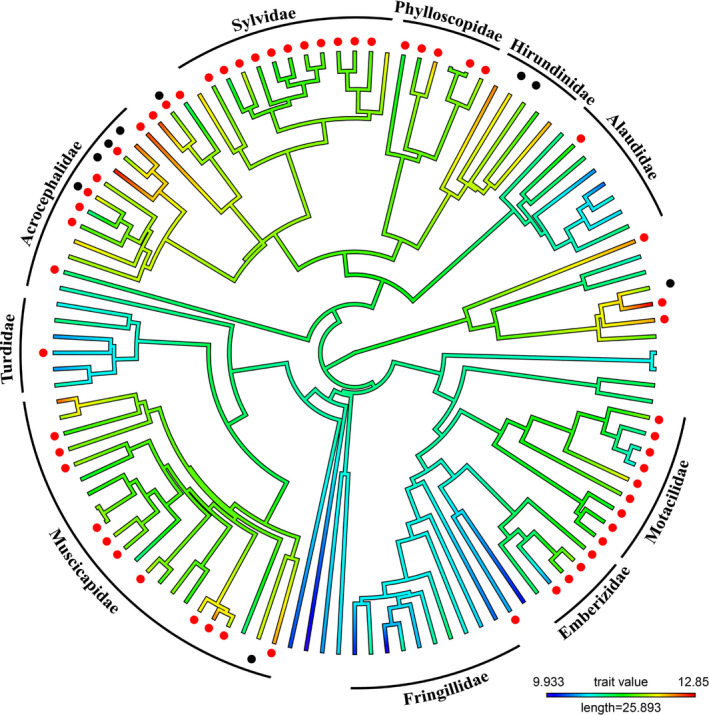
Phylogeny of the 116 species and subspecies of Western Palearctic passerines. The color of the branches represents the average photoperiod where red is longer photoperiod. The red dots indicate the species doing a prealternate molt while the black dots indicate a prebasic molt on the overwintering grounds. We labeled the passerine families with more than 5 members. See supporting information for phylogeny with tips labeled with species name

**FIGURE 2:**
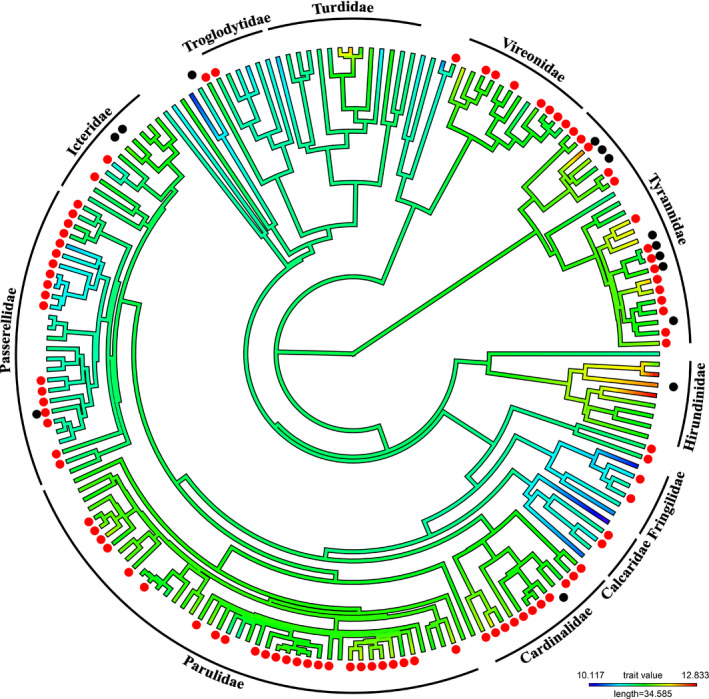
Phylogeny of the 183 species and subspecies of Nearctic passerines. The color of the branches represents the average photoperiod where red is longer photoperiod. The red dots indicate the species doing a prealternate molt while the black dots indicate a prebasic molt on the overwintering grounds. We labeled the passerine families with more than 5 members. See supporting information for phylogeny with tips labeled with species name

Using BirdTree.org (Jetz et al., 2012), we downloaded 1,000 possible trees from “Ericson All Species: a set of 10,000 trees with 9,993 OTUs each” (Ericson et al., 2006) for a phylogeny subset of the 177 Nearctic species and a subset of the 114 Western Palearctic species. Using TreeAnnotator V.1.10.4 (Rambaut & Drummond, 2018), we then created the maximum clade credibility trees for Nearctic and Palearctic with our 1,000 trees using 1% burn‐in (as states) and mean heights for node heights. We added the subspecies (6 Nearctic, 2 Palearctic) in R (R Core Team, 2020) using the package phytools (Revell, 2012) to obtain a maximum clade credibility tree of 183 species and subspecies for the Nearctic and 116 for the Western Palearctic. We used trees including the species and subspecies for all our analyses. The visual representations of the phylogenies (Figures 1 and 2) were created using the phytools package (Revell, 2012).

### 2.5 Statistical analysis

For each response variable (prebasic winter, prealternate, and complete prealternate molt), we separated the analysis between Western Palearctic and Nearctic passerines as we hypothesized that both systems are influenced differently by the environmental conditions. Note that we could not analyze the presence of a complete prealternate molt in Nearctic passerines because only one species in North America exhibits a complete prealternate molt. Prior to our analyses, we tested for collinearity; migration distance, and average photoperiod were highly correlated (*r* > 0.5) and were never included in the same model. Next, we used phylogenetic logistic linear models to test the predictors and created all the possible models (total of 23 models for prebasic molt and 47 for prealternate). We selected the best models using Akaike's information criterion (AIC) and determined that models were similar if they differed by <4 ΔAIC. The Akaike weights were obtained using the qpcR package (Spiess, 2018) and the *R* ^2^ using the function R2.lik from the rr2 package (Ives & Daijiang, 2018). Finally, we examined the 95% confidence intervals of the parameter estimates of every predictor included in the top models to assess which variables were informative. Analyses were conducted in R (R Core Team, 2020; version 1.2.5001) using the package phyloglm (Ho & Ane, 2014). The “logistic_MPLE” method was applied with a btol of 35, a log.alpha.bound of 10, and 100 bootstraps.

## 3 RESULTS

### 3.1 Prebasic winter molt

**FIGURE 3:**
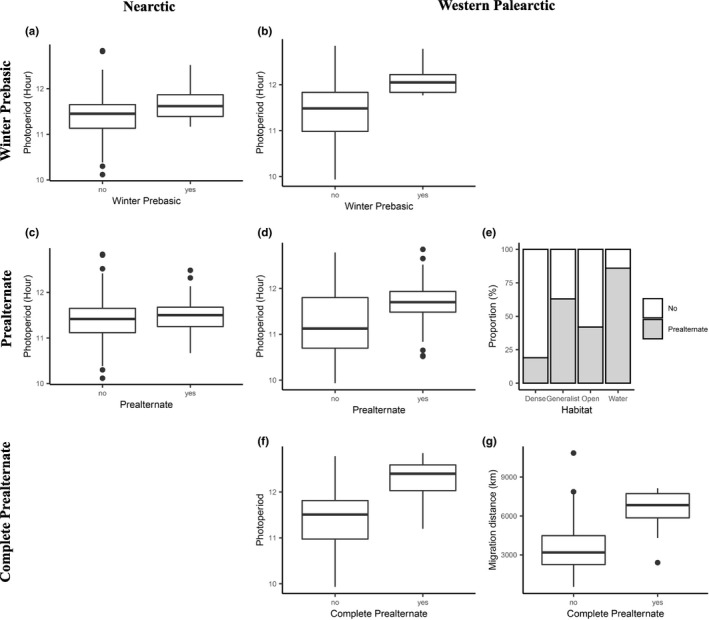
Boxplots and bar graph of the significant predictors (parameter estimates did not overlap 0) associated winter prebasic (a, b), prealternate (c, d, e), and complete prealternate molts (f, g). Plots to the left (a, c) correspond to the results for the Nearctic passerines, and plots to the right (b, d, e, g, f) are the results for the Western Palearctic species

For the Western Palearctic species, the top model predicting the presence of a prebasic winter molt included NDVI of the breeding grounds and average photoperiod on the overwintering grounds (Table 2). For the Nearctic species, the same two variables were also present in the top models, but with the addition of migration distance and NDVI of the overwintering grounds (Table 2). For both Western Palearctic and Nearctic passerines, only photoperiod had a 95% confidence interval that did not overlap zero (Table 3). The parameter estimates were both positive (Western Palearctic: 2.19, Nearctic: 1.48) which indicate that longer photoperiods were associated with a prebasic molt on the overwintering grounds in both Palearctic and Nearctic passerines (Figures 1, 2, 3).

**TABLE 2:** Top‐ranked models (<4 AIC units from top model) explaining a prebasic molt on the overwintering grounds and the presence of a second molt (prealternate), which can be completed, in Western Palearctic and Nearctic passerines

Molt	Region	Top models	AIC	ΔAIC	*w*	*R* ^2^
Prebasic winter	Western Palearctic	NDVI breed + photoperiod	52.65	0	1	0.35
	Nearctic	Photoperiod	84.12	0	0.19	0.26
		NDVI breed	85.24	1.12	0.11	0.24
		NDVI w	85.25	1.13	0.11	0.24
		Migration distance	85.35	1.23	0.10	0.24
		NDVI w + photoperiod	86.11	1.99	0.07	0.26
		NDVI w + migration dist.	87.43	3.31	0.04	0.24
		NDVI breed + migration dist.	87.54	3.42	0.03	0.24
		NDVI breed + NDVI w	87.55	3.43	0.03	0.24
		NDVI breed + NDVI w + photoperiod	88.04	3.92	0.03	0.26
Prealternate	Western Palearctic	Photoperiod + habitat w	137.84	0	0.34	0.35
		NDVI w + Photoperiod + habitat w	139.51	1.67	0.15	0.35
		NDVI breed + Photoperiod + habitat w	139.67	1.83	0.14	0.35
		Photoperiod	139.79	1.95	0.13	0.28
		NDVI w + photoperiod	139.91	2.07	0.12	0.29
		NDVI w + photoperiod + diet	141.25	3.41	0.06	0.30
		Photoperiod + diet	141.78	3.94	0.05	0.29
	Nearctic	Photoperiod	221.8	0	0.16	0.25
		Migration distance	221.8	0	0.16	0.25
		NDVI w	222	0.2	0.14	0.24
		NDVI breed + migration dist.	222.1	0.3	0.14	0.26
		NDVI breed + NDVI w + migration	223.2	1.4	0.08	0.26
		NDVI breed	223.5	1.7	0.07	0.24
		NDVI w + migration dist.	224	2.2	0.05	0.24
		NDVI w + diet	224.3	2.5	0.04	0.26
		NDVI w + photoperiod	224.4	2.6	0.03	0.24
		Migration dist. + habitat w	224.9	3.1	0.03	0.26
		Diet	225.1	3.3	0.03	0.24
		NDVI breed + NDVI w	225.2	3.4	0.03	0.24
		Habitat w	225.4	3.6	0.03	0.25
		NDVI breed + NDVI w + diet	225.6	3.8	0.02	0.26
		NDVI breed + migration dist. + diet	225.8	4	0.02	0.26
Complete prealternate	Western Palearctic	NDVI breed + photoperiod	45.39	0	0.31	0.52
		NDVI breed + migration dist.	46.49	1.1	0.18	0.51
		Migration distance	47.25	1.86	0.12	0.46
		NDVI breed + NDVI w + photoperiod	47.47	2.08	0.11	0.52
		Photoperiod	47.64	2.25	0.10	0.46
		NDVI breed + photoperiod + habitat w	48.48	3.09	0.07	0.57
		NDVI w + migration dist.	48.62	3.23	0.06	0.48
		NDVI w + photoperiod	48.70	3.31	0.06	0.47

Note

NDVI breed = NDVI breeding grounds, NDVI w = NDVI overwintering grounds, migration distance = migration distance between the centroid of the breeding and overwintering grounds, photoperiod = average photoperiod at the centroid of the wintering ground between 15 September and 15 April, habitat w = main habitat used on the overwintering grounds, diet = principal diet of the species (omnivore, invertivore, herbivore).

**TABLE 3:** Model‐averaged parameter estimates and 95% confidence intervals for variables included in the top‐ranked models (<4 AICc units of best model) explaining a prebasic molt on the overwintering grounds and the presence of a second molt (prealternate), which can be completed, in Western Palearctic and Nearctic passerines

	Prebasic winter		Prealternate		Prealternate completed
	Western Palearctic	Nearctic	Western Palearctic	Nearctic	Western Palearctic
NDVI breed	−3.39 (−6.61, 1.10)	0.001 (−2.21, 2.88)	−0.94 (−2.74, 0.028)	−0.32 (−1.66, 1.18)	4.63 (−0.002, 10.1)
NDVI w		0.44 (−2.86, 3.35)	0.80 (−1.40, 3.30)	1.83 (−0.14, 4.26)	4.98 (−0.65, 9.30)
Migration distance		0.021 (−0.30, 0.26)		0.14 (−0.070, 0.33)	**0.21, (0.10, 0.90)**
Photoperiod	**2.19 (2.11, 2.30)**	**1.48 (1.44, 1.64)**	**1.29 (1.21, 1.36)**	**0.50, (0.44, 0.59)**	**3.12 (3.02, 3.24)**
Diet—Invertivore			−0.059 (−1.00, 0.74)	0.21 (−0.61, 0.98)	
Diet—Omnivore			−0.28 (−1.21, 0.58)	0.051 (−0.89, 0.93)	
Habitat Generalist			**1.37 (0.64, 2.60)**	0.0066 (−0.55, 0.64)	0.22 (−0.48, 1.45)
Habitat—Open			0.51 (−0.62, 1.71)	−0.15 (−1.08, 0.61)	0.083 (−1.05, 0.96)
Habitat—Water			**1.97 (0.28, 3.74)**	0.75 (−0.30, 1.88)	0.13 (−13.55, 1.16)

Note

Values in bold indicate that the 95% CI did not overlap zero. See Table 2 for variable's definition.

### 3.2 Presence of a prealternate molt

The top models explaining the presence of a prealternate molt in Western Palearctic passerines included the following variables: NDVI of the breeding and overwintering grounds, photoperiod, habitat of the overwintering grounds, and diet (Table 2). Of these five variables, the confidence intervals of average photoperiod, generalist habitat, and water habitat did not overlap zero (Table 3). The parameter estimates of average photoperiod (1.29), generalist habitat (1.37), and water habitat (1.97) all indicated a positive association with prealternate molt; longer photoperiod, species wintering in water habitat or being generalist in their selection of habitat were associated with the presence of a prealternate molt (Figures 1 and 3). For the Nearctic passerines, the top models contained all the variables (NDVI breeding and overwintering grounds, migration distance, photoperiod, habitat of the overwintering grounds, and diet), but only the confidence intervals of photoperiod did not overlap zero (Tables 2 and 3). Longer photoperiod (0.50) was associated with the presence of a prealternate molt in Nearctic passerines (Figures 2 and 3).

### 3.3 Presence of a complete prealternate molt

Western Palearctic passerines that underwent a complete prealternate molt had top models that included the following variables: NDVI of the breeding and overwintering grounds, migration distance, photoperiod, and habitat of the overwintering grounds (Table 2). Only migration distance and photoperiod had a 95% confidence interval that did not overlap zero (Table 3). The parameter estimate of migration distance and photoperiod was both positive (0.21 and 3.12), suggesting that longer migration distance and longer day length are associated with the presence of a complete prealternate molt (Figure 3).

## 4 DISCUSSION

The aim of this study was to explore the potential drivers of the evolution of winter molts in Nearctic and Western Palearctic passerines. We examined the prebasic molt on the overwintering grounds, the presence of a prealternate molt (second molt) on the overwintering grounds, and the presence of a complete prealternate molt. We found that a prebasic molt on the overwintering grounds is associated with longer average photoperiods on the overwintering grounds for Nearctic as well as Western Palearctic passerines. Our results also indicate, for Western Palearctic passerines, that longer photoperiods on the overwintering grounds and species living in water (wetlands or marine) habitats or being generalist in their habitat choice exhibit an association with the presence of prealternate molt. For Nearctic passerines, only longer photoperiods were associated with the prealternate molt. Finally, the complete prealternate molt in Western Palearctic passerines is associated with longer migration distance and longer photoperiod on the overwintering grounds. Collectively, these results indicate the powerful selective force of overwintering conditions in the evolution of molt strategies.

The prebasic molt on the overwintering grounds for both Nearctic and Western Palearctic migrants is influenced by longer photoperiod on the overwintering grounds. This result was unexpected because we predicted that longer migration distance (Kjellén, 1994; Leu & Thompson, 2002) and primary productivity (Barta et al., 2008; Remisiewicz et al., 2019) would be associated with winter prebasic molt. However, photoperiod and migration distance were correlated (see Methods), making these two factors difficult to disentangle. Additionally, we were expecting longer photoperiods to be a potential driver of the prealternate molt and not the prebasic molt because we hypothesized that longer photoperiods would expose the feathers to UV radiation and, as a consequence, faster feather degradation which would be counterproductive to a prebasic molt on the overwintering grounds. However, longer photoperiods could have some benefits by reducing the costs associated with molt by increasing the time window when birds can absorb essential nutrients for molt (Murphy & King, 1991; Renfrew et al., 2011). Thus, some species might have evolved a winter prebasic molt strategy to take advantage of the longer photoperiods on the overwintering grounds compared with shorter daylights in fall on the summer grounds. We could not add diet in our models for the prebasic molt as most bird (78%) undertaking a winter prebasic molt has a diet that mostly consists of invertebrates (e.g., *Empidonax* spp., *Hippolais* spp.). Despite being unable to include this factor in our analysis, we argue that this relationship likely indicates a link between diet and winter prebasic molt. A link between diet and molt strategy has already been observed by Froehlich et al. (2005), but for the prealternate molt, long‐distance migrants with an extensive plant diet are more likely to undergo a prealternate molt for Nearctic passerines.

The evolution of the prealternate molt in Nearctic and Western Palearctic migrants is proposed to result from the amount of feather degradation caused by the habitat where they overwinter (Pyle & Kayhart, 2010; Rohwer et al., 2005; Terrill et al., 2020). In overwintering grounds with longer days throughout the winter, both Nearctic and Western Palearctic passerines may have evolved prealternate molts to cope with increased feather wear from UV light exposure (Barta et al. 2008; Bergman, 1982; Pyle, 1998). For Western Palearctic migrant passerines, species who inhabited water habitats, or were generalists, were more likely to undergo a prealternate molt. This result was counter to our hypothesis that species living in open habitats were more likely to undergo a prealternate molt because the harsher conditions and exposure to UV light would increase feather wear (Rohwer et al., 2005). Feather replacement is energetically costly (Murphy & King, 1992; Lindström et al., 1993) and requires sufficient resources, particularly proteins (Froehlich et al., 2005). Thus, species living in habitats with no or little food limitation might be able to afford the replacement of some or all feathers before spring migration. Extended UV light exposure from longer photoperiods coupled with resources availability could have led to Western Palearctic passerines evolving a second molt during their annual cycle. Habitat was not a significant variable for Nearctic passerines; these passerines generally overwinter in tropical habitats with softer foliage which does not damage feathers (Rohwer et al., 2005). In summary, the prealternate molt, which is often incomplete, seems to be associated with species affected by strong feather wear and need to replace those specific feathers to maximize fitness. This result supports the findings of Terrill et al. (2020) which identified feather wear as a driver of the evolution of prealternate molt in Parulidae, but not of Guallar and Figuerola (2016) that identified migration distance as a predominant factor and sexual selection as a limited factor in Motacillidae.

Interestingly, it appears that in most cases where winter prebasic molt is present (13 species in the Nearctic and 9 species in the Western Palearctic), a prealternate molt is uncommon (36% in Nearctic, 33% in Palearctic). Having a later prebasic molt on the overwintering grounds could result in fresher feathers prior to the spring migration and breeding season, making it unnecessary to molt feathers a second time in the spring. The effect of a prebasic molt on the presence or absence of a prealternate molt should be explored further.

The presence of a complete prealternate molt in Western Palearctic passerines appears to be associated with species with longer migration distance and longer photoperiod on the overwintering grounds. This result fits with our predictions that a complete prealternate molt would have evolved in species wintering in harsher and sun‐exposed environments (longer photoperiod and open habitat types) which damage the feathers faster, hence the need to replace them in a second molt (Jenni & Winkler, 2020; Jones, 1995; Rohwer et al., 2005). Even though we did not find an association with habitat type, longer photoperiod on the overwintering grounds could cause the need to molt before spring migration due to rapid degradation of the feathers from UV exposure (Bergman, 1982; Jenni & Winkler, 2020, see studies by Svensson & Hedenström, 1999; Jiguet et al., 2019). However, it is difficult to disentangle the effects of migration distance and photoperiod as these variables are highly correlated. Still, having to migrate a long distance could require a fresh molt prior to it to ensure feathers of high quality for the migration or longer migration distances could cause more feather wear and create the need to replace the feathers a second time. Figuerola and Jovani (2001) also identified longer migration distance as an important factor associated with prealternate molt in Western Palearctic species while Guallar and Figuerola (2016) found this association in North American Motacillidae. We could not repeat the complete prealternate molt analysis with Nearctic passerines because only one species (*Dolichonyx oryzivorus*; Renfrew et al., 2011) that breeds in North America undergoes a complete prealternate molt. Future analyses on the full range of prealternate molt extents (not just presence/absence of complete molt), as recently undertaken on preformative molts by several recent studies (e.g., Guallar et al., 2021), could be informative.

We had hypothesized that NDVI could be an important predictor of molting strategies on the overwintering grounds, but NDVI was not a significant predictor of any molt strategy. This lack of relationship could be explained by our choice of averaging the NDVI of the overwintering grounds over a period of 7 months (15 September to 15 April). This decision could have reduced the importance of certain environmental events and reduced the importance of NDVI as a potential driver in our results. It is difficult to select a precise time window that suits every species as molt timing can be variable and timing is often unknown. However, it could be interesting for future studies to separate the NDVI during the overwintering months in multiple time periods that cover the earlier prebasic molt (September–December) and the later prealternate molt (January–April).

Molt is a complex process that affects birds for multiple seasons and impacts many aspects of their life such as flight, thermoregulation, communication, and sexual selection (Gill, 2006). Therefore, the evolution of molting strategies was likely influenced by multiple variables impacting at least one function of the plumage. In this study, we focused on the importance of having a fresh plumage of high quality, but we did not examine the role of sexual selection on molt strategies, particularly prealternate molt. For future work, it would be important to examine feather coloration and degree of sexual dichromatism, especially as it may play an important role in the evolution of prealternate molt which generally happens before spring migration and breeding. Terrill et al. (2020) found that, in Parulidae, seasonal dichromatism can only evolve when a prealternate molt already exists. Froehlich et al. (2005) hypothesized that, if the cost of a second molt is too high, birds might keep their conspicuous plumage throughout the winter even though this strategy may be maladaptive. Thus, it would be interesting to examine the relation between dichromatism and prealternate molt across all passerines. Another factor that would have been interesting to analyze is the impact of molt duration on molt strategies (De la Hera et al., 2012); however, current available information on this subject is limited. It would also be interesting to extend the Tökölyi et al. (2008) study to all passerines and test breeding onset in relation to the presence of the prealternate molt.

One challenge with this study was the classification of molt strategies. Many species (e.g., *Tyrannus vociferans*) have interindividual variation or plasticity in molt. In addition, there is variation in which types of feathers and how many are molted, especially for the prealternate molt. As a consequence, a simple binary classification does not capture the complexity of molt even though this approach was necessary due to methodological limitations. To fully understand the biological complexity of winter molts, it will be necessary for future studies to categorize the extent of molt as a continuous variable (e.g., Table 3, Jenni & Winkler, 2020).

In conclusion, our results suggest that the evolution of winter molt strategies in Nearctic and Western Palearctic migratory passerines was likely driven by multiple factors, but photoperiod, migration distance, and overwintering ground conditions are particularly important. What remains to be seen is how the availability of resources, and their influence on the costs of feather production and plumage quality, has played a role in these systems. It is highly important to understand the drivers behind the different molting strategies because the quality of the molt will impact fitness throughout the annual cycle (Dawson et al., 2000; Harrison et al., 2011; Nilsson & Svensson, 1996). There is a need for a full annual cycle focus in animal ecology to effectively conserve populations (Marra et al., 2015). In that context, our findings indicate that rapid changes in conditions on the overwintering grounds (e.g., through climate change and/or habitat loss) could have substantial impacts on the selective forces shaping molt strategies, potentially requiring populations to have sufficient plasticity or adaptive capacity to overcome impacts on survival and reproduction. Alternatively, the strong role of static components of the abiotic environment, such as photoperiod, in the evolution of molt strategies may preclude some species from responding to changing biotic conditions, with unknown consequences for fitness.

## CONFLICT OF INTEREST

We declare we have no conflict of interests.

## AUTHOR CONTRIBUTIONS

**Claudie Pageau:** Conceptualization (equal); data curation (lead); formal analysis (lead); funding acquisition (equal); methodology (equal); visualization (equal); writing‐original draft (lead); writing‐review & editing (equal). **Jared Sonnleitner:** Conceptualization (supporting); data curation (equal); formal analysis (supporting); methodology (supporting); writing‐original draft (supporting); writing‐review & editing (supporting). **Christopher M. Tonra:** Conceptualization (equal); data curation (equal); investigation (equal); methodology (equal); supervision (equal); validation (equal); writing‐original draft (equal); writing‐review & editing (equal). **Mateen Shaikh:** Formal analysis (equal); methodology (equal); software (equal); supervision (equal); validation (equal); visualization (equal); writing‐original draft (supporting); writing‐review & editing (supporting). **Matthew W. Reudink:** Conceptualization (equal); data curation (equal); formal analysis (equal); funding acquisition (equal); investigation (equal); methodology (equal); resources (equal); supervision (equal); validation (equal); visualization (equal); writing‐original draft (equal); writing‐review & editing (equal).

## Supporting information

Supplementary Material

Supplementary Material

## Data Availability

Data are accessible on Dryad (https://doi.org/10.5061/dryad.5dv41ns6b).
