# Supplementary figures and images for: Evolution of winter molting strategies in European and North American migratory passerines
Source: Ecol Evol. 2021 Sep 1;11(19):13247–58. doi: 10.1002/ece3.8047 (PMC8495808; doi:10.1002/ece3.8047)

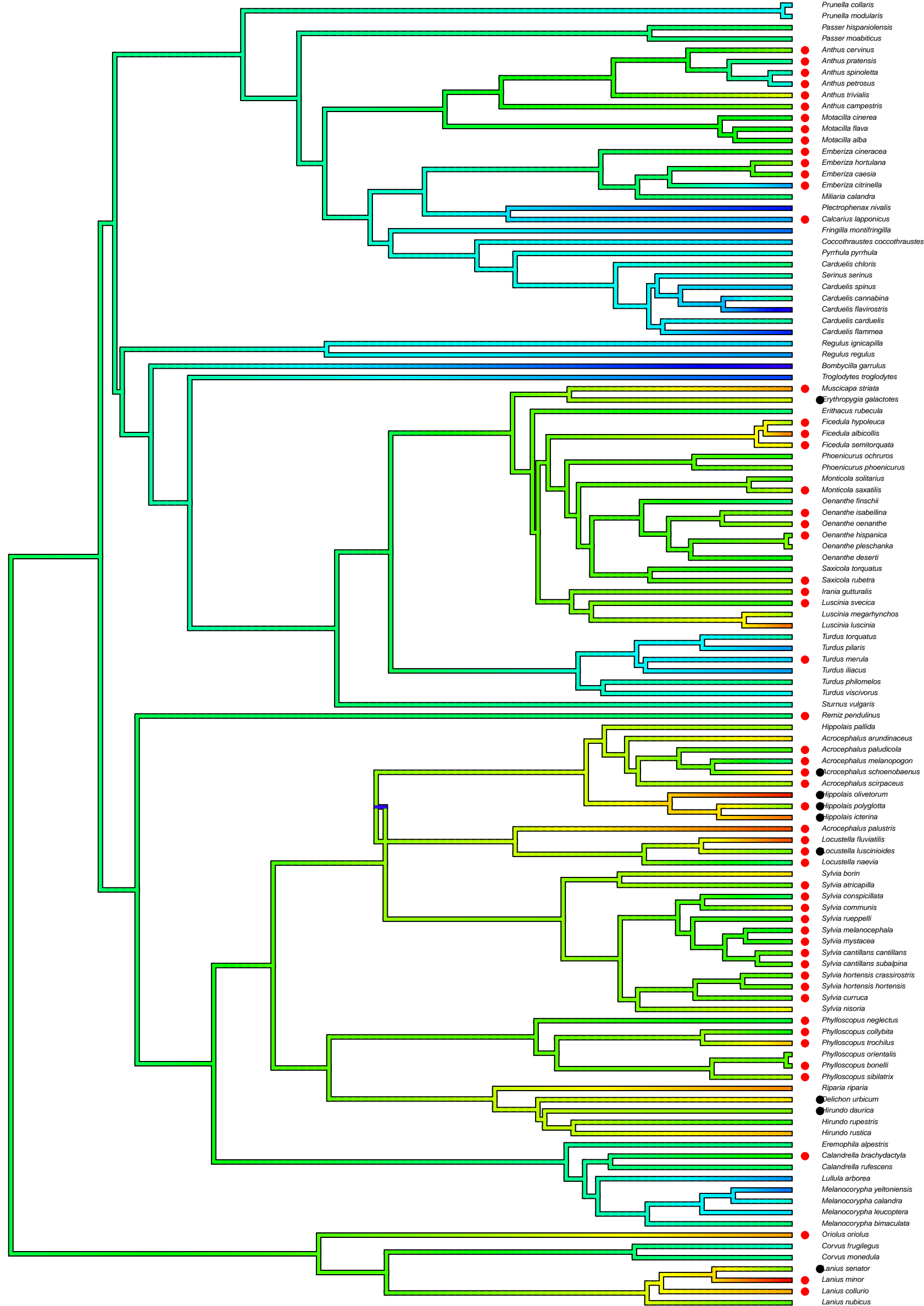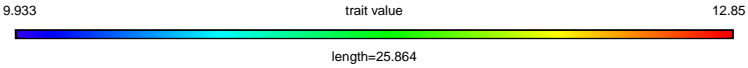

Supplement: Supplementary file 1 — Supplementary Material [file ECE3-11-13247-s001.pdf]

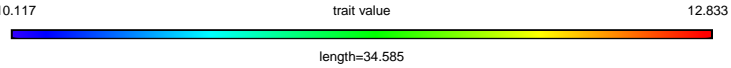

Supplement: Supplementary file 2 — Supplementary Material [file ECE3-11-13247-s002.pdf]
